# Prediction of genomic breeding values for growth, carcass and meat quality traits in a multi-breed sheep population using a HD SNP chip

**DOI:** 10.1186/s12863-017-0476-8

**Published:** 2017-01-26

**Authors:** Luiz F. Brito, Shannon M. Clarke, John C. McEwan, Stephen P. Miller, Natalie K. Pickering, Wendy E. Bain, Ken G. Dodds, Mehdi Sargolzaei, Flávio S. Schenkel

**Affiliations:** 1 Centre for Genetic Improvement of Livestock, University of Guelph, Guelph, N1G2W1 Canada; 2 AgResearch, Invermay Agricultural Centre, Private Bag 50034, Mosgiel, 9053 New Zealand; 3 Focus Genetics, Napier, 4110 New Zealand; 4 The Semex Alliance, Guelph, N1H6J2 Canada

**Keywords:** GBLUP, Ovine HD SNP Chip, Genomic selection, Eating quality traits

## Abstract

**Background:**

New Zealand has some unique Terminal Sire composite sheep breeds, which were developed in the last three decades to meet commercial needs. These composite breeds were developed based on crossing various Terminal Sire and Maternal breeds and, therefore, present high genetic diversity compared to other sheep breeds. Their breeding programs are focused on improving carcass and meat quality traits. There is an interest from the industry to implement genomic selection in this population to increase the rates of genetic gain. Therefore, the main objectives of this study were to determine the accuracy of predicted genomic breeding values for various growth, carcass and meat quality traits using a HD SNP chip and to evaluate alternative genomic relationship matrices, validation designs and genomic prediction scenarios. A large multi-breed population (*n* = 14,845) was genotyped with the HD SNP chip (600 K) and phenotypes were collected for a variety of traits.

**Results:**

The average observed accuracies (± SD) for traits measured in the live animal, carcass, and, meat quality traits ranged from 0.18 ± 0.07 to 0.33 ± 0.10, 0.28 ± 0.09 to 0.55 ± 0.05 and 0.21 ± 0.07 to 0.36 ± 0.08, respectively, depending on the scenario/method used in the genomic predictions. When accounting for population stratification by adjusting for 2, 4 or 6 principal components (PCs) the observed accuracies of molecular breeding values (mBVs) decreased or kept constant for all traits. The mBVs observed accuracies when fitting both **G** and **A** matrices were similar to fitting only **G** matrix. The lowest accuracies were observed for k-means cross-validation and forward validation performed within each k-means cluster.

**Conclusions:**

The accuracies observed in this study support the feasibility of genomic selection for growth, carcass and meat quality traits in New Zealand Terminal Sire breeds using the Ovine HD SNP chip. There was a clear advantage on using a mixed training population instead of performing analyzes per genomic clusters. In order to perform genomic predictions per breed group, genotyping more animals is recommended to increase the size of the training population within each group and the genetic relationship between training and validation populations. The different scenarios evaluated in this study will help geneticists and breeders to make wiser decisions in their breeding programs.

**Electronic supplementary material:**

The online version of this article (doi:10.1186/s12863-017-0476-8) contains supplementary material, which is available to authorized users.

## Background

The New Zealand meat sheep industry plays a very important role in the international market, being the third largest sheep meat producer [1]. In 2015 the country produced 488,000 tonnes of sheep meat with 98% available for export to a variety of countries (e.g. China, United Kingdom and United States of America) [2]. Well-designed breeding programs have sustained industry competitiveness, with substantial genetic progress in several traits of high economic relevance (e.g. increase of 83% in kg of lamb produced per ewe and up to 28% overall in carcass weight from 1990 to 2012, [3]). Increased production efficiency is directly related to profitability. However, to maintain this change and to increase the proportion entering the premium markets, both meat presentation and quality have to be improved continuously. Historically, this has included the use of electrical stimulation in post slaughter, and a shift from frozen to chilled product primarily improving tenderness. In addition to tenderness, other meat quality traits now should be also incorporated into breeding programs in order to further genetically improve or maintain the meat quality. It is a challenge to improve meat quality traits by traditional breeding methods due to the fact that most of these traits are expensive to measure and may require slaughter of the potential selection candidates. Progeny testing implies additional costs for the producers and an increase in generation interval, which limits genetic gains per year that could be achieved if progenitors were selected early in life. Genomic selection (GS) [4] is revolutionising livestock breeding programs worldwide and is one of the most promising tools to genetically improve quality and production of sheep meat.

Genomic predictions for a number of standard production traits are already implemented in the New Zealand and worldwide sheep industries [5–10]. New Zealand has some unique genetic resources that include Terminal Sire composite breeds which were developed in the last three decades to meet commercial needs. These composite breeds include Primera, Lamb Supreme, Landmark and Highlander composites. As reported by Brito [11] and Kijas et al. [12], these composites and the breeds involved in their formation have high genetic diversity and large effective population sizes (N_e_). For instance, N_e_ of 974, 380 and 227 have been reported for Primera, Lamb Supreme and Texel breed groups, respectively [11]. N_e_ is negatively related to levels of linkage disequilibrium, which is an important factor to successfully predict molecular breeding values [13]. Therefore, to enable GS in the New Zealand Terminal Sire composite breeds, a high density SNP array (606,006 SNPs) was commissioned by FarmIQ™ (joint New Zealand government and industry Primary Growth Partnership) and developed in conjunction with the International Sheep Genomics Consortium (ISGC) and Illumina [14, 15]. The availability of a higher density panel could be a great option to successfully conduct multi-breed genomic evaluations and make faster genetic progress in the traits of interest (e.g. growth, carcass and meat quality traits).

Furthermore, it is important to investigate the best methods/scenarios for genomic predictions in these populations. When there is a close relationship between the animals in the training and validation population, molecular breeding values (mBVs) can be estimated with a higher accuracy [16]. Ventura et al. [17], in a study with beef cattle, has proposed a method to improve genomic selection by clustering animals based on their genotype information. The idea was to create groups of animals that are more genetically similar so that SNP effects would be consistent within these clusters and therefore improve accuracy of genomic predictions. However, this methodology has not been evaluated in sheep populations yet and could be beneficial for the population under investigation due to its high genetic diversity.

Accounting for population structure can also be an important step in genomic analysis. In a sheep study, Auvray et al. [6] fitted six principal components (PCs) from the decomposition of the centered genotype matrix as fixed effects in the mBVs estimation model to account for population structure and Dodds et al. [18] also evaluated this strategy by fitting PCs from the genomic relationship matrix in the genomic predictions in a Dual-purpose sheep population. Considering that, it is also important to evaluate the need to adjust for population structure in the Terminal Sire composite breeds under investigation, due to the fact that this is a unique population, with some genetic connectedness among the breed groups and common ancestral breeds.

The main objectives of this study were to determine the accuracy of genomic predictions of breeding values for various growth, carcass and meat quality traits using a HD SNP chip and to evaluate alternative genomic relationship matrices, validation designs and genomic prediction scenarios.

## Methods

### Genotype data and quality control

There were 14,845 animals from both sexes (7961 males and 6884 females) with HD (Ovine Infinium® HD SNP Beadchip) genotype call rate greater than 95%. The animals were born in: 2007–2009 (*n* = 208); 2010 (*n* = 3623); 2011 (*n* = 3782), 2012 (*n* = 2383), 2013 (*n* = 2175) and 2014 (*n* = 2674). DNA was extracted mostly from ear punch tissue, however, DNA was also extracted from blood and semen samples [19–21]. Genotyping was conducted at the AgResearch Animal Genomics Research Laboratory, Mosgiel, New Zealand.

Genotypes were called on the AB system and using Illumina GenomeStudio® software. Genotypes were coded as the number of A alleles (0, 1 or 2). SNPs were excluded from the analysis if minor allele frequency (MAF) was less than 0.01, call rate less than 0.95, non-autosomal markers, unknown genomic position on OARv3.1, had duplicated map positions (two SNP with the same position but with different names), misplaced SNP positions compared to the sheep reference genome assembly version OARV3.1 or an extreme departure from Hardy Weinberg equilibrium (HWE, *p* < 10^−15^). A total of 517,902 SNP were retained for further analyses after filtering. Following quality control, missing genotypes were minimal (2.16%) and were imputed using the FImpute software [22].

### Phenotypic data

Performance records were obtained from the Sheep Improvement Limited (SIL, www.sil.co.nz) database. Only animals that were genotyped with the HD SNP chip and measured for at least one trait were included in this investigation, as the main goal was to estimate prediction accuracies of molecular breeding values. Performance records were obtained from 14,845 animals born between 2007 and 2014 (progeny birth years: 2010 to 2014, sire birth years: 2007 to 2013) in the FarmIQ, Ram Breeding and Progeny Test flocks. Farms (*n* = 6) were located on the North and South Islands of New Zealand. The animals were primarily progeny from Terminal Sire composites and Texels mated to a variety of maternal/dual-purpose breeds. Progeny data from 877 rams were included in this study. The average (± SD) number of progeny per sire was 17 (±15) and it ranged from 1 to 114 progeny per sire.

### Traits description and data editing

The traits included in this study were: birth weight (BWT, kg), weaning weight (WWT, kg), live weight at 6 months (LW6, kg), eye muscle depth (EMD, mm), eye muscle width (EMW, mm) and fat depth (FDM, mm) measured by ultrasound, pre-slaughter weight (PRESLT, kg) measured around 24 h prior to slaughter, hot carcass weight (HCW, kg), cold carcass weight (CCWT, kg), dressing out percentage (DO%, %) estimated as: $\frac{HCW}{PRESLT}\ *100$, X-ray carcass weight (XWT), X-ray leg weight (XLEG, kg), X-ray middle or loin weight (XMID, kg) and X-ray forequarter weight (XFORE, kg), X-ray number of rib pairs (XNRIB, n), depth of tissue at the GR site over the 12^th^ rib at a distance of 110 mm from mid-line (CGRM, mm), carcass measurement of buttocks circumference (CBUTT, cm), loin meat pH (LPH), meat colour measures indicated by L*n* (lightness/darkness), A*n* (redness/brownness) and B*n* (yellowness), with *n* being 24, 48, 96 and 168 h after retail display, marbling score (MARB, visually scored on a five point scale) and shear force as an indicator of tenderness (SHF, kg). A detailed description of the traits evaluated and its recording procedures can be found in Brito [11].

Data handling and preparation were performed predominantly in R [23]. Only records that met the following criteria were used: 1) animal genotyped with HD SNP chip; 2) year of birth and birth flock known; 3) sex identified as male or female, 4) trait management group known and 5) contemporary group (CG) for the trait contained more than three observations. To remove possible outliers, observations more than three standard deviations outside the mean for the contemporary group, were also deleted. Contemporary group is trait specific and was defined by flock, birth year, sex, weaning mob (except for birth weight) and trait measurement mob.

### Expected accuracy of genomic predictions

The expected accuracies (AccE) were estimated as the correlation between true and estimated genomic values, i.e. $\sqrt{\frac{N_p{h}^2}{N_p{h}^2 + {M}_e}}$ [24], where *N*_*p*_ is the number of individuals in the training population (genotyped and measured for each trait), h^2^ is the trait heritability and M_e_ is the effective number of loci, which can be calculated as 2N_e_L/log(4N_e_L) [25], where assumed genome length (*L*) was 26 Morgans [8].

### Effective number of progeny

The EBV of a young lamb for a trait for which it has no phenotype record is based on the information of its relatives. Using genomic information, it is possible to generate a breeding value at an earlier age with an accuracy higher than the parent average. One could be interested in knowing the number of progeny that would need to be recorded to achieve an EBV’s accuracy similar to the one attained by using genomic information. Therefore, we defined Effective Number of Progeny (ENP) as the number of progeny needed to complement the parent average information to yield the same accuracy as the mBVs. ENP has been previously reported in sheep studies [19] and it was calculated using the formula: ENP = (r^2^α)/(1 ‐ r^2^), where r^2^ is mBVs reliability, ∝ = (4 − h^2^)/h^2^, and h^2^ is the trait heritability.

### Genomic BLUP (prediction of molecular breeding values)

The phenotype fitted in the models for estimation of SNP effects were the phenotypes adjusted for known systematic and contemporary group effects that affects individual records (same models used to estimate heritability but excluding the animal effect). The effects were determined in a previous study using the same dataset [11]. The software snp1101 [26] was used for the analyses. The mBVs were calculated for each trait based on the following mixed model:

$$ \boldsymbol{y} = 1\upmu +\boldsymbol{W}\boldsymbol{a}+\boldsymbol{e} $$

where ***y*** is the vector of observed phenotypic values of the animals adjusted for fixed effects (Additional file 1), **1** is a vector of 1 s, μ is the overall mean, **W** is the design matrix linking records to animal mBVs, **a** is the vector of random animal mBVs and **e** is the vector of random residual effects. The mBVs were assumed normally distributed with mean zero and variance equal to $\mathbf{G}*{\sigma}_{\fontfamily{Calibri Light}{g}}^2$, where **G** is the genomic relationship matrix based on the SNP markers and ${\sigma}_{\fontfamily{Calibri Light}{g}}^2$ is the genetic variance. The random residual effects were assumed normally distributed with mean zero and variance equal to **I** * *σ*_*e*_^2^, where **I** is an identity matrix and *σ*_*e*_^2^ is the residual variance. The mBVs are the predicted animal effects from the above model and corresponds to the sum of the effects of each SNP. The effect of three different versions of **G** on accuracy of mBVs were investigated:

1. **G matrix as in VanRaden** [27]**:** The **G** matrix was calculated as: $\boldsymbol{G} = \frac{\left(\boldsymbol{M}-2\boldsymbol{P}\right){\left(\boldsymbol{M}-2\boldsymbol{P}\right)}^{\prime}}{2{\displaystyle \sum }{\boldsymbol{p}}_{\boldsymbol{i}}\left(1-{\boldsymbol{p}}_{\boldsymbol{i}}\right)}$, where **M** is a matrix of counts of the alleles “A”, *p*_*i*_ is the frequency of allele “A” of the i^th^ SNP, **P** is a matrix with each row containing the *p*_*i*_ values. Missing values in **M** were imputed using the software FImpute [22]. Hereafter, this **G** matrix will be described as **GB0**.
2. **G + A matrices:** an alternative **G** matrix was fitted as G* = (1 - ***w***)**G** + ***w*** **A**, where **G** is the genomic relationship matrix **GB0** and **A** is the pedigree relationship matrix. Attributing a weight (***w***) for **A** is equivalent to fitting residual polygenic effects that are not captured by the markers [28, 29]. Three weights were evaluated: w = 0, 10 and 20. Hereafter these will be described as **GB0** (same as the one previously described), **GB10** and **GB20**, respectively.
3. **Genomic predictions using G calculated based on base population allele frequencies (GBBP):** According to VanRaden [27], allele frequencies from the unselected population should be used to construct the **G** matrix. The effects of calculating the **G** matrix based on the allele frequencies of the base population was evaluated. This method has been implemented in the software snp1101 [26] and is based on a modified version of Colleau indirect algorithm [30].

### Accounting for population structure

To determine whether accounting for population structure would increase the accuracy of genomic predictions, phenotypes where adjusted for fixed effects (as described previously) and for two (**GB2PC**), four (**GB4PC**), or six (**GB6PC**) covariate principal components from the genomic relationship matrix.

### Validation designs

For each individual trait the total number of records were split into training and validation populations to a) derive a prediction equation of performance based on HD SNP genotypes using the training population and b) to estimate the accuracy of the prediction equation in the validation population. The validation scenarios evaluated were:

1. **Forward validation and mixed training population:** for each trait, all animals with genotypes and phenotypes were split into two populations based on birth year: training (birth years: 2007 to 2013) and validation (birth year: 2014) populations. The youngest cohort of animals were used in validation to mimic what would happen in practice (young animals without phenotypes recorded would be selected based on marker effects predicted on older animals). **GB0**, **GB2PC**, **GB4PC**, **GB6PC**, **GB10**, **GB20** and **GBBP** were compared using this validation scenario.
2. **Forward validation within each k-means cluster (GBC):** the animals were clustered in five groups as explained later in the section “k-means clustering”. The animals from each cluster were then divided into two groups: training (birth years: 2007 to 2013) and validation (birth year: 2014) populations to perform genomic predictions. The mean accuracy for all the groups was weighted by the number of records in the validation population within each group.
3. **Forward validation within each genomic cluster:** following Ventura et al. [17], we evaluated different clustering methodologies based solely on genotype information. After clustering, the animals from each cluster were treated as an independent population and genomic predictions were conducted within each group (i.e. cluster) using forward validation (split in training and validation populations as described before). The clustering methodologies evaluated were based on a distance matrix built based on: 1) Genomic relationship matrix (**GB0**) [27], and 2) Euclidean genotype distance matrix (**EDM**) [31]. Hierarchical clusters were determined using the *hclust* package in R [23]. The animals from each cluster were then divided into two groups: training (birth years: 2007 to 2013) and validation (birth year: 2014) populations. The mean accuracy was weighted by the number of records in the validation population. K*n*G and K*n*EDM represents these scenarios, where *n* is the number of assumed subpopulations and **G** and **EDM** represents the information used to build the distance matrices used for clustering the animals.
4. **Cross-validation:** The data was divided into five datasets and each subset is predicted once from the other subsets. The prediction equations were derived from four groups and validated in the 5^th^ group. It was alternated until all groups were used as validation. The genomic prediction accuracies were considered as the average of the five analysis. The dataset was divided based on two procedures:
  - **Randomly (GBRCV):** each animal was randomly assigned to one of five subsets.
  - **k-means clustering (GBKCV):** similar to Saatchi et al. [32], the animals were also clustered based on the k-means clustering approach, based on Hartigan and Wong’ algorithm [33]. The distance matrix was created based on the genomic relationship matrix (**GB0**) among genotyped animals [27]. The choice for five groups was based on i) the plot of the first two principal components and ii) that the majority of animals with records were born from 2010 to 2014 (5 years), which could potentially balance the number of animals per group and facilitate the comparisons with the other scenarios.

### Accuracies of genomic predictions

The observed accuracy of mBVs were derived, for each validation population, as the Pearson correlation between mBVs and phenotypes (adjusted for fixed effects or also fitting principal components of **GB0** matrix). The Pearson correlation was then divided by the square root of heritability (h^2^) to adjust for the upper limit of accuracy of a phenotype/residual (*y*) $\left( r\left( mBVs,\  y\right)/\sqrt{h^2}\right)$. The heritability was estimated from the same dataset using Restricted Maximum Likelihood (REML) procedures fitting an animal model and the same fixed effects described before (Additional file 1), using ASReml [34]. The pedigree was recorded since 1990 and contained 243,486 individuals. Accuracies were reported only when the number of individuals (in the validation population) was greater than 150. When combining accuracies across breed groups or clusters, the overall accuracy was the mean of the accuracy within each group weighted by the number of records.

As presented in VanRaden et al. [27, 35], from the inverse of the left hand side of the mixed model equations (MME) it is possible to calculate theoretical accuracy (**AccT**) of the estimated genomic values. This accuracy has practical application to sheep producers, as it gives a measure of the mBV accuracy for each individual animal that is candidate to selection.

### Spread of molecular breeding values

Following Dodds et al. [18], the spread of mBVs in the validation populations were examined to make sure they were consistent with what was expected for a set of estimated breeding values with mean accuracy *r*. Given that the accuracies of the mBVs are constant: $v a r\left(\frac{mBV{s}^{*}}{r}\right) = \frac{var\left( mBV{s}^{*}\right)}{r^2}= v a r(TBV)={\sigma}_u^2$, where “^*^” denotes the mBVs adjusted to have the correct variance as: ${r}^2=\frac{var\left( mBV{s}^{*}\right)}{var(TBV)}$. From this, the factor ***K***, by which the mBVs must be multiplied to have the right spread, can be calculated as: mBVs* = *k* * mBVs*. Furthermore, $v a r\left(\frac{K* mBV{s}^{*}}{r}\right) = \frac{K^2 var(mBVs)}{r^2}= v a r(TBV)$ and $K = \frac{r* sd(TBV)}{sd(mBVs)}$. Considering that, the ratio of the expected spread to that observed was measured as: *K* = *r* * *σ*_*A*_/*sd*(*mBV*), where *σ*_*A*_^2^ is the genetic variance of the trait and *sd(mBV)* is the standard deviation of the mBVs for the trait.

## Results

Table 1 summarizes all phenotypic traits based on the following parameters: number of observations, mean, standard deviation and phenotypic range for all growth, carcass and meat quality traits. The difference in number of records is because only genotyped animals were included in this study and not all of them were measured for all the traits, plus some traits were not recorded in all flocks (e.g. BWT) and a quality control of the raw data was done as previously described. The size of training and validation populations for all genomic prediction scenarios is presented in Additional file 2. The average (± SD) number of animals in the training population was 8519 ± 2009 (GB0, GB2PC, GB4PC, GB6PC, GBBP, GB10 and GB20), 8538 ± 1868 (GBRCV and GBKCV), 1706 ± 397 (GBC), 8400 ± 1960 (K5EDM), 8502 ± 2017 (K5G), 8271 ± 1925 (K10EDM) and 4223 ± 1091 (K10G). Heritability estimates for traits measured in the live animal, carcass and meat quality traits ranged from 0.10 to 0.43 (average: 0.28 ± 0.08), 0.14 to 0.28 (average: 0.22 ± 0.03) and 0.04 to 0.31 (average: 0.16 ± 0.07), respectively.

**Table 1:** Descriptive statistics for growth, carcass and meat quality traits

Trait (measurement unit)	Abbreviation	N	Mean ± SD	Range
Traits measured in the live animal				
Birth weight^a^, kg	BWT	1206	4.97 ± 1.01	2.1–8.0
Weaning weight, kg	WWT	14,781	31.64 ± 6.00	14.0–51.8
Live weight at 6 months, kg	LW6	14,146	38.47 ± 6.38	19.6–60.5
Pre-slaughter weight, kg	PRESLT	13,744	41.95 ± 6.55	23.6–61.0
Ultrasonic eye muscle depth, mm	EMD	7838	25.44 ± 2.87	18.0–35.0
Ultrasonic eye muscle width, mm	EMW	7853	65.94 ± 6.03	49.0–86.0
Ultrasonic fat depth, mm	FDM	7767	2.72 ± 1.12	0.0–5.0
Carcass traits				
Hot carcass weight, kg	CWT	13,750	18.04 ± 3.35	7.1–27.9
Cold carcass weight, kg	CWTC	13,702	17.59 ± 3.27	8.2–27.4
Dressing out percentage, %	DO%	13,727	42.96 ± 3.09	33.7–52.8
Butt circumference, cm	CBUTT	13,698	65.22 ± 3.30	54.8–75.0
GR^b^, mm	CGRM	13,698	5.48 ± 3.62	0.0–18.0
X-ray weight, kg	SFWT	13,398	17.49 ± 3.29	7.7–27.66
X-ray leg weight, kg	SFLEG	13,212	6.07 ± 1.04	2.98–9.34
X-ray middle weight, kg	SFMID	13,210	5.37 ± 1.14	2.03–8.94
X-ray number of rib pairs	SFRIB	13,289	13.01 ± 0.33	12–14
X-ray fore weight, kg	SFFORE	13,228	6.00 ± 1.17	2.65–9.62
Meat quality traits				
Loin meat pH	LPH	10,241	5.80 ± 0.17	5.45–6.40
Marbling score	MARB	10,617	3.12 ± 0.59	1–5
Tenderness score	SHF	10,255	6.40 ± 2.14	1.45–12.99
CIE a* after 24 h	A24	10,472	17.41 ± 2.79	9.62–26.8
CIE a* after 48 h	A48	10,472	15.56 ± 2.27	9.06–23.82
CIE a* after 96 h	A96	10,470	13.11 ± 2.06	6.77–19.79
CIE a* after 168 h	A168	10,105	10.87 ± 2.23	2.25–20.8
CIE b* after 24 h	B24	10,445	13.64 ± 2.89	4.87–20.08
CIE b* after 48 h	B48	10,415	12.78 ± 2.59	4.86–18.57
CIE b* after 96 h	B96	10,444	12.06 ± 2.47	4.74–17.56
CIE b* after 168 h	B168	9992	10.78 ± 2.75	3.5–17.03
CIE L* after 24 h	L24	10,134	39.32 ± 3.94	28.79–51.93
CIE L* after 48 h	L48	10,135	39.33 ± 3.90	29.07–51.46
CIE L* after 96 h	L96	10,145	39.44 ± 3.91	29.29–51.75
CIE L* after 168 h	L168	9830	39.09 ± 4.04	28.79–52.06

^a^: trait measured in a reduced number of flocks; ^b^: Depth of tissue 110 mm off the mid-line in the region of the 12^th^ rib; *N* number of observations; *SD* standard deviation

### Accuracies of genomic predictions

**Fig. 1:**
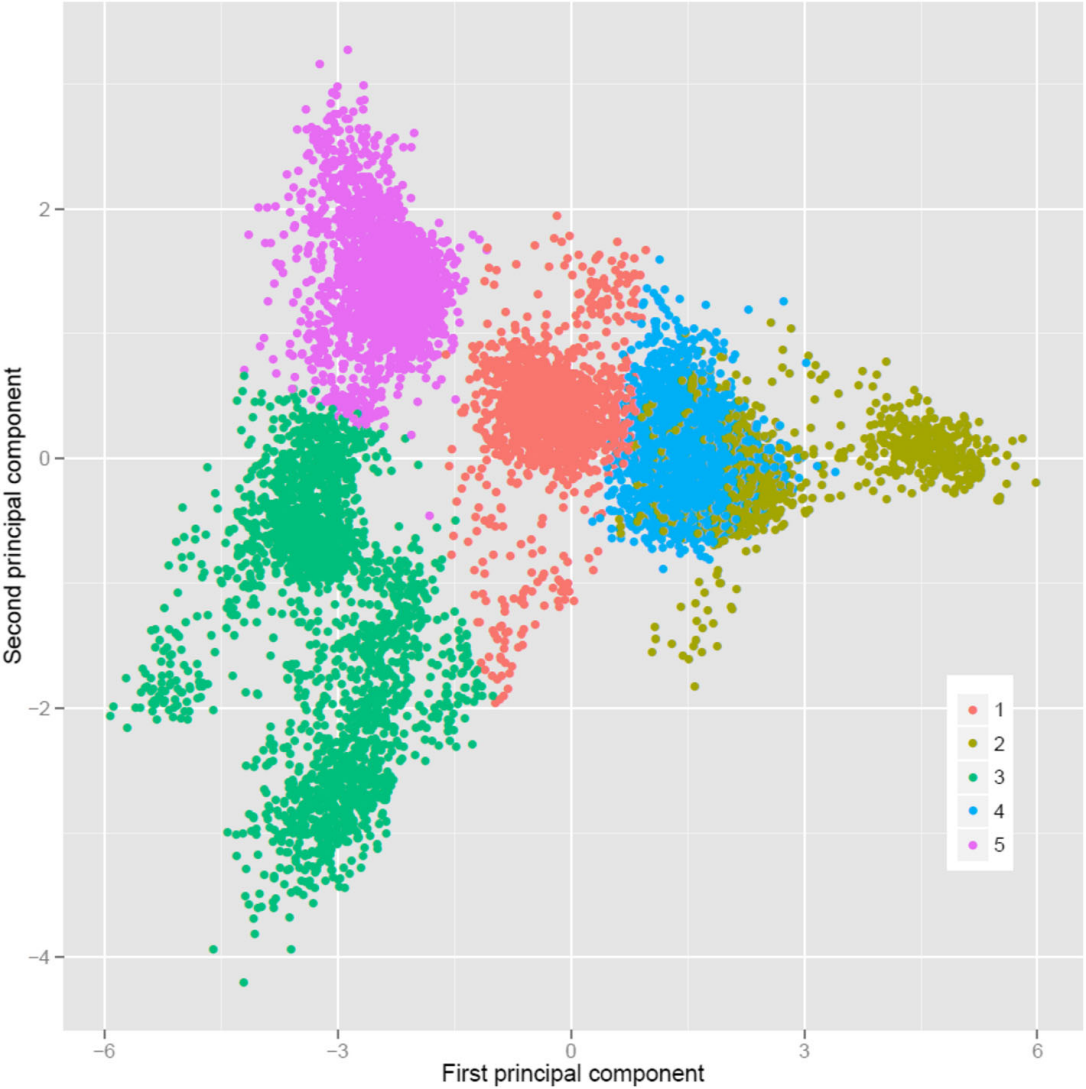
Principal component decomposition of the genomic relationship matrix colored by k-means clusters

The accuracies of genomic predictions for GB0, GB2PC, GB4PC, GB6PC, GB10, GB20, GBRCV, GBKCV and GBC are presented in Tables 2, 3 and 4 for traits measured in the live animal, carcass traits and meat quality traits, respectively. The expected average accuracies (AccE) for traits measured in the live animal, carcass traits and meat quality traits were 0.41 ± 0.11, 0.46 ± 0.03 and 0.34 ± 0.07, respectively. The average observed accuracies (± SD) for traits measured in the live animal for the scenarios GB0, GB2PC, GB4PC, GB6PC, GB10, GB20, GBRVC, GBKCV and GBC were 0.33 ± 0.10, 0.28 ± 0.09, 0.28 ± 0.10, 0.27 ± 0.10, 0.33 ± 0.09, 0.33 ± 0.09, 0.48 ± 0.06, 0.26 ± 0.07 and 0.18 ± 0.07, respectively. For carcass traits the average observed accuracies (± SD) were 0.50 ± 0.08, 0.42 ± 0.10, 0.40 ± 0.09, 0.36 ± 0.08, 0.50 ± 0.09, 0.51 ± 0.09, 0.55 ± 0.05, 0.33 ± 0.05 and 0.28 ± 0.09, respectively. And lastly, for the meat quality traits the average observed accuracies (± SD) were 0.29 ± 0.10, 0.27 ± 0.11, 0.28 ± 0.10, 0.26 ± 0.11, 0.29 ± 0.11, 0.28 ± 0.11, 0.36 ± 0.08, 0.21 ± 0.07 and 0.23 ± 0.06, respectively. The number of animals clustered in each of the five groups using k-means approach was 1485, 1590, 2570, 6706 and 2494 animals in cluster 1, 2, 3, 4 and 5, respectively (Fig. 1). The average *ENP* (± SD) was 2.00 ± 0.74, 6.58 ± 2.46 and 2.83 ± 1.11 for traits measured in the live animal, carcass traits and meat quality traits, respectively. The traits that required the greater number of progeny to attain similar accuracies of those using genomic data were carcass traits, followed by meat quality traits and then traits measured in the live animal.

**Table 2:** Heritability estimates, expected accuracy, theoretical accuracy, effective number of progeny and observed accuracies of molecular breeding values in different scenarios for traits measured in the live animal (growth and carcass traits)

Trait^1^	h^2^ ± SE	AccE	AccT	ENP	GB0	GB2PC	GB4PC	GB6PC	GB10	GB20	GBRCV	GBKCV	GBC
BWT	0.10 ± 0.03	0.08	0.25	1	0.15	0.14	0.14	0.12	0.16	0.18	0.33 ± 0.24	0.14 ± 0.06	0.11 ± 0.02
WWT	0.19 ± 0.02	0.45	0.45	1	0.19	0.17	0.15	0.14	0.19	0.19	0.46 ± 0.01	0.24 ± 0.08	0.08 ± 0.06
LW6	0.30 ± 0.01	0.51	0.48	1	0.25	0.23	0.22	0.21	0.25	0.25	0.41 ± 0.03	0.22 ± 0.06	0.14 ± 0.09
EMD	0.35 ± 0.01	0.44	0.49	2	0.40	0.37	0.38	0.37	0.40	0.40	0.53 ± 0.03	0.28 ± 0.05	0.20 ± 0.08
EMDad	0.43 ± 0.01	0.48	0.46	3	0.48	0.43	0.44	0.44	0.48	0.48	0.56 ± 0.01	0.37 ± 0.06	0.24 ± 0.11
EMW	0.24 ± 0.01	0.38	0.45	2	0.31	0.31	0.32	0.30	0.31	0.31	0.52 ± 0.03	0.28 ± 0.05	0.23 ± 0.13
EMWad	0.31 ± 0.01	0.42	0.47	3	0.44	0.43	0.43	0.42	0.44	0.43	0.54 ± 0.02	0.33 ± 0.06	0.31 ± 0.12
FDM	0.28 ± 0.01	0.41	0.45	2	0.33	0.23	0.22	0.22	0.33	0.33	0.48 ± 0.04	0.24 ± 0.08	0.16 ± 0.02
FDMad	0.33 ± 0.01	0.43	0.47	2	0.37	0.23	0.22	0.20	0.37	0.38	0.49 ± 0.04	0.27 ± 0.10	0.15 ± 0.10
PRESLT	0.25 ± 0.02	0.49	0.44	3	0.35	0.28	0.25	0.25	0.35	0.35	0.47 ± 0.02	0.28 ± 0.09	0.16 ± 0.11
Average	0.28 ± 0.08	0.41 ± 0.11	0.44	2.00 ± 0.74	0.33 ± 0.10	0.28 ± 0.09	0.28 ± 0.10	0.27 ± 0.10	0.33 ± 0.09	0.33 ± 0.09	0.48 ± 0.06	0.26 ± 0.07	0.18 ± 0.07

^1^Abbreviations are presented in Table 1; “ad”: traits that were adjusted for correlated variables; h^2^: heritability estimate; AccE: Expected accuracy; AccT: Theoretical accuracy from the MME; ENP: effective number of progeny calculated using the accuracies from GB0; GB0: GBLUP accuracies fitting only G matrix; GB10 and GB20: accuracies of GBLUP fitting G matrix and 10 or 20% of A matrix, respectively; GB2PC, GB4PC and GB6PC: GBLUP accuracies fitting for 2, 4 or 6 principal components, respectively; GBRCV: GBLUP accuracies for random cross-validation; GBKCV: GBLUP accuracies for k-means clustering; GBC: GBLUP accuracies for predictions performed within each cluster

**Table 3:** Heritability estimates, expected accuracy, theoretical accuracy, effective number of progeny and observed accuracies of molecular breeding values in different scenarios for carcass traits

Trait^1^	h^2^ ± SE	AccE	AccT	ENP	GB0	GB2PC	GB4PC	GB6PC	GB10	GB20	GBRCV	GBKCV	GBC
CCWT	0.23 ± 0.02	0.48	0.42	4	0.44	0.38	0.35	0.31	0.44	0.44	0.51 ± 0.01	0.30 ± 0.08	0.22 ± 0.16
HCWT	0.21 ± 0.02	0.46	0.42	5	0.46	0.40	0.37	0.33	0.46	0.46	0.52 ± 0.01	0.31 ± 0.08	0.23 ± 0.17
SFXWT	0.19 ± 0.02	0.44	0.41	6	0.47	0.40	0.36	0.32	0.47	0.47	0.54 ± 0.02	0.30 ± 0.12	0.22 ± 0.16
DRESS	0.24 ± 0.02	0.48	0.46	13	0.67	0.66	0.66	0.61	0.67	0.67	0.67 ± 0.02	0.47 ± 0.07	0.51 ± 0.10
CBUTT	0.28 ± 0.02	0.51	0.45	5	0.52	0.51	0.46	0.40	0.53	0.53	0.55 ± 0.02	0.37 ± 0.07	0.31 ± 0.18
CBUTTad	0.24 ± 0.02	0.48	0.46	9	0.59	0.56	0.52	0.46	0.60	0.61	0.60 ± 0.02	0.37 ± 0.04	0.45 ± 0.14
CGRM	0.23 ± 0.02	0.47	0.43	7	0.54	0.32	0.32	0.33	0.55	0.55	0.55 ± 0.02	0.28 ± 0.07	0.26 ± 0.09
CGRMad	0.23 ± 0.02	0.47	0.48	8	0.57	0.34	0.34	0.31	0.58	0.58	0.59 ± 0.01	0.35 ± 0.07	0.35 ± 0.17
SFFORE	0.17 ± 0.02	0.41	0.39	6	0.45	0.42	0.37	0.32	0.45	0.45	0.53 ± 0.03	0.32 ± 0.14	0.23 ± 0.12
SFLEG	0.18 ± 0.02	0.42	0.39	8	0.52	0.44	0.42	0.36	0.52	0.52	0.54 ± 0.03	0.32 ± 0.10	0.25 ± 0.17
SFMID	0.24 ± 0.02	0.47	0.44	5	0.49	0.34	0.34	0.31	0.49	0.49	0.51 ± 0.02	0.28 ± 0.11	0.14 ± 0.12
SFRIB	0.14 ± 0.02	0.38	0.35	3	0.30	0.30	0.30	0.30	0.30	0.30	0.44 ± 0.03	0.31 ± 0.06	0.22 ± 0.21
Average	0.22 ± 0.03	0.46 ± 0.03	0.43	6.58 ± 2.46	0.50 ± 0.08	0.42 ± 0.10	0.40 ± 0.09	0.36 ± 0.08	0.50 ± 0.09	0.51 ± 0.09	0.55 ± 0.05	0.33 ± 0.05	0.28 ± 0.09

^1^Abbreviations are presented in Table 1; “ad”: traits that were adjusted for correlated variables; h^2^: heritability estimate; AccE: Expected accuracy; AccT: Theoretical accuracy from the MME; ENP: effective number of progeny calculated using the accuracies from GB0; GB0: GBLUP accuracies fitting only G matrix; GB10 and GB20: accuracies of GBLUP fitting G matrix and 10 or 20% of A matrix, respectively; GB2PC, GB4PC and GB6PC: GBLUP accuracies fitting for 2, 4 or 6 principal components, respectively; GBRCV: GBLUP accuracies for random cross-validation; GBKCV: GBLUP accuracies for k-means clustering; GBC: GBLUP accuracies for predictions performed within each cluster

**Table 4:** Heritability estimates, expected accuracy, theoretical accuracy, effective number of progeny and observed accuracies of molecular breeding values in different scenarios for meat quality traits

Trait^1^	h^2^ ± SE	AccE	AccT	ENP	GB0	GB2PC	GB4PC	GB6PC	GB10	GB20	GBRCV	GBKCV	GBC
A24	0.17 ± 0.02	0.36	0.39	3	0.31	0.31	0.30	0.30	0.31	0.30	0.37 ± 0.07	0.24 ± 0.04	0.20 ± 0.07
A24ad	0.16 ± 0.02	0.35	0.38	4	0.35	0.34	0.35	0.35	0.34	0.33	0.37 ± 0.06	0.22 ± 0.08	0.26 ± 0.08
A48	0.17 ± 0.02	0.36	0.35	2	0.23	0.22	0.22	0.21	0.22	0.21	0.38 ± 0.02	0.27 ± 0.05	0.14 ± 0.10
A48ad	0.17 ± 0.02	0.36	0.37	2	0.26	0.25	0.25	0.25	0.26	0.25	0.40 ± 0.02	0.30 ± 0.06	0.16 ± 0.10
A96	0.19 ± 0.02	0.38	0.36	2	0.23	0.22	0.21	0.19	0.24	0.24	0.35 ± 0.03	0.19 ± 0.06	0.11 ± 0.10
A96ad	0.18 ± 0.02	0.37	0.38	2	0.26	0.25	0.25	0.24	0.26	0.26	0.37 ± 0.03	0.23 ± 0.05	0.16 ± 0.11
A168	0.06 ± 0.02	0.22	0.20	1	0.02	−0.04	0.06	−0.06	0.02	0.03	0.19 ± 0.05	0.07 ± 0.07	0.21 ± 0.14
A168ad	0.06 ± 0.02	0.22	0.15	1	0.08	−0.02	0.03	−0.03	0.08	0.09	0.25 ± 0.04	0.12 ± 0.10	0.19 ± 0.11
B24	0.14 ± 0.02	0.33	0.28	3	0.29	0.28	0.28	0.28	0.29	0.29	0.33 ± 0.04	0.18 ± 0.05	0.22 ± 0.13
B48	0.13 ± 0.02	0.33	0.34	2	0.24	0.23	0.23	0.23	0.24	0.24	0.29 ± 0.03	0.16 ± 0.05	0.19 ± 0.11
B96	0.13 ± 0.02	0.32	0.35	3	0.29	0.29	0.29	0.28	0.28	0.28	0.34 ± 0.04	0.24 ± 0.08	0.23 ± 0.07
B168	0.14 ± 0.02	0.32	0.28	4	0.32	0.30	0.31	0.30	0.32	0.31	0.35 ± 0.05	0.24 ± 0.06	0.23 ± 0.06
L24	0.18 ± 0.02	0.37	0.38	3	0.32	0.31	0.31	0.31	0.32	0.32	0.42 ± 0.03	0.22 ± 0.04	0.28 ± 0.07
L48	0.20 ± 0.02	0.39	0.39	2	0.31	0.31	0.32	0.32	0.31	0.31	0.39 ± 0.03	0.24 ± 0.06	0.26 ± 0.10
L96	0.21 ± 0.02	0.40	0.39	3	0.33	0.31	0.32	0.32	0.33	0.33	0.41 ± 0.04	0.23 ± 0.06	0.25 ± 0.11
L168	0.20 ± 0.02	0.38	0.38	3	0.33	0.32	0.32	0.32	0.34	0.34	0.43 ± 0.05	0.22 ± 0.05	0.26 ± 0.14
SHF	0.26 ± 0.03	0.43	0.41	2	0.28	0.28	0.27	0.27	0.28	0.28	0.41 ± 0.03	0.27 ± 0.05	0.19 ± 0.09
SHFad	0.27 ± 0.03	0.44	0.44	2	0.30	0.29	0.29	0.28	0.30	0.30	0.41 ± 0.03	0.27 ± 0.05	0.18 ± 0.11
MARB	0.31 ± 0.03	0.47	0.44	5	0.52	0.43	0.43	0.43	0.52	0.52	0.50 ± 0.03	0.35 ± 0.03	0.33 ± 0.14
MARBad	0.31 ± 0.03	0.47	0.45	5	0.52	0.46	0.46	0.45	0.52	0.53	0.52 ± 0.03	0.36 ± 0.03	0.36 ± 0.13
LPH	0.14 ± 0.02	0.33	0.32	4	0.33	0.32	0.31	0.29	0.33	0.33	0.27 ± 0.03	0.15 ± 0.07	0.20 ± 0.07
LPHad	0.13 ± 0.02	0.32	0.31	4	0.34	0.32	0.32	0.29	0.34	0.34	0.27 ± 0.03	0.15 ± 0.08	0.20 ± 0.06
Average	0.16 ± 0.07	0.34 ± 0.07	0.35	2.83 ± 1.11	0.29 ± 0.10	0.27 ± 0.11	0.28 ± 0.10	0.26 ± 0.11	0.29 ± 0.11	0.28 ± 0.11	0.36 ± 0.08	0.21 ± 0.07	0.23 ± 0.06

^1^Abbreviations are presented in Table 1; “ad”: traits that were adjusted for correlated variables; h^2^: heritability estimate; AccE: Expected accuracy; AccT: Theoretical accuracy from the MME; ENP: effective number of progeny calculated using the accuracies from GB0; GB0: GBLUP accuracies fitting only G matrix; GB10 and GB20: accuracies of GBLUP fitting G matrix and 10 or 20% of A matrix, respectively; GB2PC, GB4PC and GB6PC: GBLUP accuracies fitting for 2, 4 or 6 principal components, respectively; GBRCV: GBLUP accuracies for random cross-validation; GBKCV: GBLUP accuracies for k-means clustering; GBC: GBLUP accuracies for predictions performed within each cluster

VanRaden [27] proposed that **G** should be calculated using the allele frequencies from the base population. However, in this study there were no differences in accuracies of genomic predictions when using the observed or base population allele frequencies (GBBP versus GB0). Therefore, accuracies for GBBP were not presented separately.

**Fig. 2:**
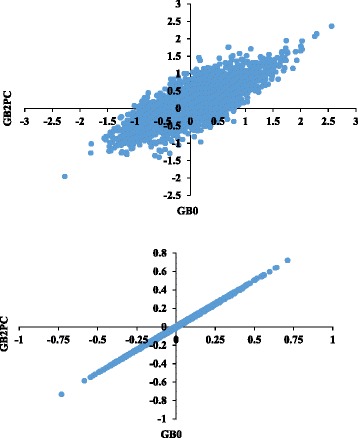
Molecular breeding values (mBVs) adjusted for 2 Principal Components of G matrix versus mBVs not adjusted for PC for the traits GR and meat redness (A24), respectively

When accounting for population stratification by adjusting for two, four or six PCs the accuracies of mBVs decreased or kept constant for all traits, with exception of some meat color traits that presented an increase of 0.01 in observed accuracy compared to GB0 (not fitting PCs). Additional file 3 presents Pearson correlations between mBVs estimated using adjusted phenotypes (not including PCs, GB0) and phenotypes also adjusted for two, four or six PCs (GB2PC, GB4PC, GB6PC, respectively). For all the traits the correlations were greater than 0.90, except for CGRM and CGRMad (0.80 and 0.75, respectively). Figure 2 shows the relationship between GB2PC and GB0 for the traits CGRM and A24 (lowest and highest Pearson correlation, respectively). In general, meat quality traits were least affected when adjusted for PCs. The average correlation between mBVs not fitting PCs or fitting two, four or six was: 0.96 ± 0.04, 0.94 ± 0.04 and 0.93 ± 0.04, respectively.

The mBVs accuracies when fitting both **G** and **A** matrix (GB10 and GB20) were similar to fitting only **G** matrix (GB0). The highest increase in accuracy was observed for BWT (0.03). The highest accuracies among all validation scenarios were observed for random cross-validation (GBRCV). The lowest accuracies were observed for k-means cross-validation (GBKCV) and forward validation performed within each k-means cluster (GBC). Even though the average accuracies for GBC were lower, there were some groups with accuracies similar to GB0. This variation in accuracies between groups/clusters is also indicated by the high standard deviation.

Table 5 presents the number of animals grouped in each cluster based on distance matrices built using **EDM** or **G** matrices and assuming number of subpopulations equal to 2, 3, 4, 5, 10 and 20. From k = 2 to 5 the majority of the animals were grouped in the same cluster. When considering k = 10 and 20, the majority of the animals were still clustered together using **EDM** approach and using **G** there was a higher variation, but still the majority of the animals were grouped in two clusters. As recommended by Ventura et al. [17] the groups with few animals could be added to the genetically closest group. In our case, doing this would mean to include almost all the animals in the same analysis (similar to GB0). Therefore, the few animals from different clusters were excluded from the analysis to evaluate the impact of excluding those less related animals. Genomic predictions were performed for all assumed number of subpopulations (2, 3, 4, 5, 10 and 20). However, they were similar and only the results for k = 5 and k = 10 were presented in this paper. The average accuracies of mBVs for these scenarios were presented in Table 6 (average for trait groups) and Additional file 4 (individual traits). Average accuracies of mBVs for K5EDM and K5G were equal to those from GB0 for all trait groups. The size of training and validation populations were also similar as few animals were clustered separately from the main cluster. For K10EDM and K10G, the average accuracies were smaller than those from GB0.

**Table 5:** Number of animals in each group divided based on clustering approaches using Euclidean Distance Matrix (EDM) or distance matrix built from G matrix

	K = 2		K = 3		K = 4		K = 5		K = 10		K = 20	
	EDM	G	EDM	G	EDM	G	EDM	G	EDM	G	EDM	G
Cluster 1	14,797	14,844	14,609	14,842	14,609	14,740	14,609	14,740	14,345	9452	13,258	9452
Cluster 2	48	1	188	2	120	102	120	102	261	4966	666	3825
Cluster 3			48	1	68	2	68	1	120	316	261	1125
Cluster 4					48	1	33	1	37	102	306	230
Cluster 5							15	1	33	2	114	102
Other									49	7	240	111

*K* number of assumed subpopulations

**Table 6:** Average observed accuracies of molecular breeding values group of animals clustered based on Euclidean Distance Matrix (EDM) or distance matrix built from G matrix and average ratio, *K*, of expected (assuming accuracies of molecular breeding values for each scenario) spread to observed spread of molecular breeding values

Trait group^1^	K5EDM		K5G		K10EDM		K10G^a^		GB0	
	Acc	*K*	Acc	*K*	Acc	*K*	Acc	*K*	Acc	*K*
Traits measured in the live animal	0.33 ± 0.11	0.82 ± 0.17	0.33 ± 0.10	0.82 ± 0.16	0.33 ± 0.10	0.84 ± 0.22	0.26 ± 0.14	0.87 ± 0.53	0.33 ± 0.10	0.81 ± 0.16
Meat quality traits	0.29 ± 0.10	0.92 ± 0.22	0.29 ± 0.11	0.90 ± 0.23	0.29 ± 0.11	0.97 ± 0.36	0.26 ± 0.08	1.06 ± 0.29	0.29 ± 0.11	0.91 ± 0.23
Carcass traits	0.50 ± 0.09	1.07 ± 0.13	0.50 ± 0.09	1.07 ± 0.13	0.49 ± 0.09	1.17 ± 0.16	0.37 ± 0.10	1.15 ± 0.27	0.50 ± 0.09	1.07 ± 0.13

^1^: Additional file 4 presents the results for individual traits; K5EDM: animals clustered based on EDM and assuming 5 subpopulations; K5G: animals clustered based on a distance matrix built from G matrix and assuming 5 subpopulations; K10EDM: animals clustered based on EDM and assuming 10 subpopulations; K10G^a^: animals clustered based on a distance matrix built from G matrix and assuming 10 subpopulations; ^a^: average for clusters 1 and 2; GB0: GBLUP accuracies fitting only G matrix; Acc: accuracy of molecular breeding values; *K*: spread of molecular breeding values

**Fig. 3:**
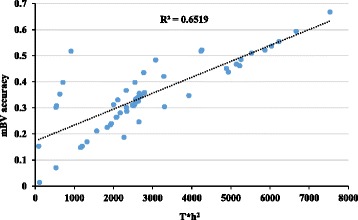
Relationship between the mBV accuracies and the number of records (T) for particular traits times heritability (h^2^, T*h^2^)

Figure 3 presents the relationship between the mBV accuracies (GB0) and the number of records (T) for particular traits times heritability (h^2^, T*h^2^), showing a linear trend (*R* ^2^ = 0.65). The average ENP for traits measured in the live animal, carcass traits and meat quality traits was 2.00 ± 0.74, 6.58 ± 2.46 and 2.83 ± 1.11, respectively (Tables 2, 3 and 4).

### Spread of molecular breeding values

As a measure of genomic inflation, Tables 6, 7, 8 and 9 present the values of *K*, which is the ratio of the expected spread in mBVs to that observed [18]. For most genomic prediction scenarios *K* was lower than 1, indicating that mBVs are more spread than expected. There was a high variation between traits and genomic prediction scenarios. The average for all the traits was: 0.93 ± 0.21, 0.87 ± 0.29, 0.89 ± 0.23, 0.83 ± 0.30, 0.97 ± 0.22, 1.02 ± 0.23, 0.92 ± 0.21, 0.50 ± 0.20, 0.40 ± 0.18, 1.02 ± 0.42, 0.93 ± 0.21, 0.92 ± 0.21, 0.99 ± 0.32, 0.82 ± 0.33, 1.05 ± 0.45 for the scenarios GB0, GB2PC, GB4PC, GB6PC, GB10, GB20, GBBP, GBRCV, GBKCV, GBC, K5EDM, K5G, K10EDM and K10G (average for cluster 1 and 2), respectively. On average, *K* values were similar among methods, except cross-validation methods that presented lower values.

**Table 7:** The ratio, *K*, of expected (assuming accuracies of molecular breeding values for each scenario) spread to observed spread of molecular breeding values for traits measured in the live animal

Trait^1^	GB0	GB2PC	GB4PC	GB6PC	GB10	GB20	GBRCV	GBKCV	GBC	k5EDM	k5G	k10EDM	K10G1	K10G2
BWT	0.66	0.66	0.61	0.56	0.72	0.80	0.36 ± 0.26	0.17 ± 0.20	0.65 ± 0.09	0.58	0.68	0.57	–	0.59
WWT	0.51	0.47	0.44	0.41	0.54	0.57	0.54 ± 0.02	0.33 ± 0.08	0.33 ± 0.29	0.53	0.55	0.52	0.27	0.28
LW6	0.64	0.60	0.59	0.56	0.67	0.71	0.55 ± 0.04	0.33 ± 0.08	0.43 ± 0.29	0.65	0.62	0.63	0.40	0.42
EMD	0.94	0.94	0.97	0.96	0.98	1.03	0.85 ± 0.07	0.51 ± 0.11	0.69 ± 0.20	0.97	0.94	0.91	0.83	1.53
EMDad	1.01	1.02	1.05	1.06	1.05	1.08	0.81 ± 0.07	0.75 ± 0.15	0.84 ± 0.33	1.02	1.02	1.16	1.09	2.54
EMW	0.86	0.88	0.91	0.85	0.90	0.93	0.77 ± 0.07	0.50 ± 0.07	0.82 ± 0.40	0.90	0.87	1.01	0.95	2.12
EMWad	1.04	1.04	1.06	1.05	1.08	1.13	0.83 ± 0.06	0.70 ± 0.10	1.23 ± 0.17	1.06	1.06	1.18	1.26	1.53
FDM	0.80	0.66	0.63	0.62	0.83	0.86	0.69 ± 0.05	0.30 ± 0.14	0.87 ± 0.15	0.82	0.80	0.78	0.75	0.05
FDMad	0.81	0.61	0.57	0.54	0.85	0.89	0.74 ± 0.07	0.38 ± 0.18	0.50 ± 0.34	0.82	0.81	0.81	0.73	0.14
PRESLT	0.82	0.71	0.66	0.64	0.87	0.92	0.62 ± 0.04	0.47 ± 0.11	0.75 ± 0.54	0.82	0.82	0.79	0.59	0.75
Average	0.81 ± 0.15	0.76 ± 0.18	0.75 ± 0.20	0.73 ± 0.21	0.85 ± 0.15	0.89 ± 0.15	0.68 ± 0.14	0.44 ± 0.16	0.71 ± 0.23	0.82 ± 0.16	0.82±	0.84 ± 0.21	0.76 ± 0.28	0.99 ± 0.79

^1^Abbreviations are presented in Table 1; “ad”: traits that were adjusted for correlated variables; h^2^: heritability estimate; GB0: spread of molecular breeding values for GBLUP fitting only G matrix; GB10 and GB20: spread of molecular breeding values for GBLUP fitting G matrix and 10 or 20% of A matrix, respectively; GB2PC, GB4PC and GB6PC: spread of molecular breeding values for GBLUP fitting 2, 4 or 6 principal components, respectively; GBRCV: spread of molecular breeding values for GBLUP and random cross-validation; GBKCV: spread of molecular breeding values for GBLUP for K-means clustering; GBC: spread of molecular breeding values for GBLUP for predictions performed within each cluster; K5EDM: spread of molecular breeding values for GBLUP when animals were clustered based on EDM and assuming 5 subpopulations; K5G: spread of molecular breeding values for GBLUP when animals were clustered based on a distance matrix built from G matrix and assuming 5 subpopulations; K10EDM: spread of molecular breeding values for GBLUP when animals were clustered based on EDM and assuming 10 subpopulations; K10G^a^: spread of molecular breeding values for GBLUP when animals were clustered based on a distance matrix built from G matrix and assuming 10 subpopulations

**Table 8:** The ratio, *K*, of expected (assuming accuracies of molecular breeding values for each scenario) spread to observed spread of molecular breeding values for carcass traits

Trait^1^	GB0	GB2PC	GB4PC	GB6PC	GB10	GB20	GBRCV	GBKCV	GBC	k5EDM	k5G	k10EDM	K10G1	K10G2
CCWT	0.94	0.86	0.84	0.78	0.98	1.03	0.59 ± 0.02	0.48 ± 0.09	1.05 ± 0.82	0.93	0.92	1.03	0.63	1.21
HCWT	0.99	0.92	0.91	0.84	1.04	1.09	0.61 ± 0.02	0.49 ± 0.08	1.09 ± 0.85	1.00	0.99	1.07	0.66	1.26
SFXWT	1.02	0.93	0.89	0.82	1.06	1.11	0.61 ± 0.03	0.48 ± 0.13	1.25 ± 1.04	1.02	1.02	1.15	0.73	1.33
DRESS	1.36	1.36	1.48	1.44	1.42	1.49	0.85 ± 0.04	0.79 ± 0.07	1.85 ± 0.33	1.37	1.36	1.54	1.45	1.77
CBUTT	1.04	1.02	1.00	0.92	1.09	1.16	0.69 ± 0.02	0.62 ± 0.05	1.06 ± 0.56	1.05	1.03	1.09	0.82	1.33
CBUTTad	1.23	1.21	1.16	1.07	1.29	1.37	0.73 ± 0.08	0.59 ± 0.03	1.39 ± 0.44	1.22	1.21	1.17	1.36	1.32
CGRM	1.03	0.78	0.82	0.84	1.07	1.13	0.62 ± 0.02	0.42 ± 0.09	0.90 ± 0.33	1.04	1.03	1.07	0.87	1.15
CGRMad	1.11	0.85	0.84	0.79	1.16	1.22	0.65 ± 0.04	0.55 ± 0.07	1.43 ± 0.84	1.11	1.11	1.24	1.03	2.34
SFFORE	1.07	1.01	0.97	0.88	1.12	1.17	0.58 ± 0.05	0.51 ± 0.15	1.43 ± 0.93	1.06	1.07	1.20	0.99	1.15
SFLEG	1.19	1.01	1.05	0.96	1.25	1.32	0.58 ± 0.03	0.51 ± 0.09	1.42 ± 0.95	1.20	1.18	1.43	0.83	1.83
SFMID	0.98	0.81	0.81	0.77	1.03	1.08	0.63 ± 0.03	0.43 ± 0.14	0.65 ± 0.63	0.98	0.98	1.01	0.54	1.02
SFRIB	0.89	0.89	0.89	0.89	0.94	0.99	0.41 ± 0.05	0.40 ± 0.07	1.34 ± 1.09	0.88	0.89	1.03	1.53	0.49
Average	1.07 ± 0.12	0.97 ± 0.16	0.97 ± 0.18	0.91 ± 0.17	1.12 ± 0.13	1.18 ± 0.13	0.63 ± 0.09	0.52 ± 0.10	1.24 ± 0.29	1.07 ± 0.12	1.07 ± 0.12	1.17 ± 0.15	0.95 ± 0.30	1.35 ± 0.42

^1^Abbreviations are presented in Table 1; “ad”: traits that were adjusted for correlated variables; h^2^: heritability estimate; GB0: spread of molecular breeding values for GBLUP fitting only G matrix; GB10 and GB20: spread of molecular breeding values for GBLUP fitting G matrix and 10 or 20% of A matrix, respectively; GB2PC, GB4PC and GB6PC: spread of molecular breeding values for GBLUP fitting 2, 4 or 6 principal components, respectively; GBRCV: spread of molecular breeding values for GBLUP and random cross-validation; GBKCV: spread of molecular breeding values for GBLUP for K-means clustering; GBC: spread of molecular breeding values for GBLUP for predictions performed within each cluster; K5EDM: spread of molecular breeding values for GBLUP when animals were clustered based on EDM and assuming 5 subpopulations; K5G: spread of molecular breeding values for GBLUP when animals were clustered based on a distance matrix built from G matrix and assuming 5 subpopulations; K10EDM: spread of molecular breeding values for GBLUP when animals were clustered based on EDM and assuming 10 subpopulations; K10G^a^: spread of molecular breeding values for GBLUP when animals were clustered based on a distance matrix built from G matrix and assuming 10 subpopulations

**Table 9:** The ratio, *K*, of expected (assuming accuracies of molecular breeding values for each scenario) spread to observed spread of molecular breeding values for meat quality traits

Trait^1^	GB0	GB2PC	GB4PC	GB6PC	GB10	GB20	GBRCV	GBKCV	GBC	k5EDM	k5G	k10EDM	K10G1	K10G2
A24	0.86	0.86	0.84	0.84	0.88	0.90	0.45 ± 0.09	0.33 ± 0.06	0.65 ± 0.28	0.88	0.87	0.78	0.62	1.01
A24ad	1.01	1.00	1.02	1.02	1.03	1.05	0.44 ± 0.09	0.32 ± 0.10	0.81 ± 0.23	1.00	1.01	0.87	0.75	1.11
A48	0.66	0.65	0.66	0.63	0.67	0.67	0.44 ± 0.03	0.41 ± 0.07	0.64 ± 0.36	0.65	0.65	0.65	0.38	1.17
A48ad	0.78	0.75	0.79	0.77	0.79	0.80	0.44 ± 0.03	0.49 ± 0.09	0.68 ± 0.36	0.77	0.77	0.82	0.57	1.29
A96	0.67	0.63	0.62	0.57	0.70	0.73	0.40 ± 0.03	0.28 ± 0.07	0.51 ± 0.40	0.65	0.66	0.65	0.31	0.79
A96ad	0.81	0.77	0.78	0.74	0.85	0.89	0.43 ± 0.04	0.35 ± 0.06	0.67 ± 0.41	0.79	0.80	0.81	0.37	0.95
A168	0.12	−0.28	0.35	−0.38	0.14	0.17	0.08 ± 0.02	0.06 ± 0.04	1.96 ± 1.42	0.16	0.10	0.22	0.24	2.49
A168ad	0.69	−0.16	0.24	−0.22	0.77	0.86	0.09 ± 0.02	0.11 ± 0.06	2.26 ± 1.41	0.73	0.66	1.27	0.63	2.97
B24	0.99	0.96	0.98	0.97	1.04	1.09	0.35 ± 0.05	0.27 ± 0.06	0.69 ± 0.67	1.00	0.99	0.92	0.57	1.31
B48	0.81	0.81	0.81	0.80	0.85	0.88	0.29 ± 0.03	0.21 ± 0.06	0.53 ± 0.46	0.84	0.81	0.80	0.58	1.30
B96	1.05	1.06	1.08	1.06	1.08	1.11	0.26 ± 0.02	0.32 ± 0.09	1.79 ± 0.59	1.07	1.04	1.41	1.15	1.78
B168	1.30	1.24	1.31	1.27	1.36	1.43	0.25 ± 0.04	0.33 ± 0.05	1.65 ± 0.59	1.31	1.30	1.79	1.59	1.21
L24	0.96	0.95	0.94	0.93	1.02	1.09	0.50 ± 0.03	0.37 ± 0.04	1.43 ± 0.16	1.01	0.96	1.06	0.75	1.54
L48	0.90	0.92	0.93	0.93	0.95	1.02	0.46 ± 0.04	0.39 ± 0.08	1.25 ± 0.68	0.90	0.90	0.91	0.60	1.61
L96	0.94	0.90	0.94	0.93	1.00	1.07	0.51 ± 0.04	0.39 ± 0.09	1.06 ± 0.48	0.94	0.94	0.90	0.55	1.50
L168	0.96	0.96	0.98	0.97	1.01	1.08	0.50 ± 0.06	0.38 ± 0.05	1.02 ± 0.43	0.97	0.96	1.08	0.77	1.42
LKGF	0.76	0.76	0.78	0.77	0.80	0.85	0.55 ± 0.04	0.43 ± 0.08	0.52 ± 0.35	0.76	0.77	0.71	0.76	0.50
LKGFad	0.80	0.80	0.80	0.78	0.85	0.90	0.58 ± 0.05	0.42 ± 0.08	0.42 ± 0.35	0.79	0.81	0.68	0.69	0.51
MARB	1.16	1.06	1.07	1.07	1.18	1.25	0.72 ± 0.05	0.62 ± 0.05	1.30 ± 0.65	1.12	1.12	1.13	1.05	1.23
MARBad	1.12	1.09	1.10	1.08	1.17	1.24	0.74 ± 0.06	0.67 ± 0.04	1.38 ± 0.58	1.12	1.12	1.17	1.09	1.33
LPH	1.08	1.05	1.00	0.94	1.15	1.23	0.22 ± 0.02	0.17 ± 0.08	1.00 ± 0.63	1.10	1.08	1.06	0.89	1.54
LPHad	1.17	1.14	1.09	1.02	1.24	1.32	0.22 ± 0.03	0.17 ± 0.09	1.02 ± 0.48	1.18	1.17	1.13	0.96	1.68
Average	0.91 ± 0.23	0.86 ± 0.33	0.90 ± 0.24	0.84 ± 0.35	0.94 ± 0.23	0.99 ± 0.25	0.40 ± 0.18	0.32 ± 0.15	1.07 ± 0.50	0.92 ± 0.22	0.90 ± 0.23	0.97 ± 0.36	0.78 ± 0.34	1.35 ± 0.53

^1^Abbreviations are presented in Table 1; “ad”: traits that were adjusted for correlated variables; h^2^: heritability estimate; GB0: spread of molecular breeding values for GBLUP fitting only G matrix; GB10 and GB20: spread of molecular breeding values for GBLUP fitting G matrix and 10 or 20% of A matrix, respectively; GB2PC, GB4PC and GB6PC: spread of molecular breeding values for GBLUP fitting 2, 4 or 6 principal components, respectively; GBRCV: spread of molecular breeding values for GBLUP and random cross-validation; GBKCV: spread of molecular breeding values for GBLUP for K-means clustering; GBC: spread of molecular breeding values for GBLUP for predictions performed within each cluster; K5EDM: spread of molecular breeding values for GBLUP when animals were clustered based on EDM and assuming 5 subpopulations; K5G: spread of molecular breeding values for GBLUP when animals were clustered based on a distance matrix built from G matrix and assuming 5 subpopulations; K10EDM: spread of molecular breeding values for GBLUP when animals were clustered based on EDM and assuming 10 subpopulations; K10G^a^: spread of molecular breeding values for GBLUP when animals were clustered based on a distance matrix built from G matrix and assuming 10 subpopulations

## Discussion

The Ovine HD SNP chip is characterized by short distance linkage disequilibrium (LD) [11] that could be enough for multi-breed genomic predictions based on LD threshold (>0.2) reported in the literature [4]. Furthermore, the consistency of gametic phase among the breed groups involved in the Terminal Sire composite breeds were high, suggesting that a mixed training population for genomic predictions could be envisioned [11]. Considering that, we conducted this study to assess the feasibility of genomic selection for a variety of growth, carcass and meat quality traits in a Terminal Sire composite population. In addition, we investigate different **G** matrices and genomic prediction validation scenarios. These scenarios were chosen to cover the best and worst case situations for genomic predictions that could happen in practice, for instance, selection on younger animals (forward validation), selection within groups (split based on genomic clusters), and selection candidates born in a range of years and in more genetically related or distant group of animals (random or k-means cross-validation, respectively).

### Genomic prediction scenarios

#### Different genomic relationship matrices

The accuracies observed for most scenarios and traits indicate that genomic selection is a very important tool to increase the rate of genetic gains in the New Zealand Terminal Sire composite sheep population. Among the forward validation scenarios, GB0 presented the highest average accuracies and is the recommended scenario for genomic predictions in this population. Accuracies for GBBP and GB0 were the same, probably because there are not many founding animals genotyped in this population (i.e. all animals genotyped were born after 2007 and the majority from 2010 to 2014) and, therefore, the allele frequency from base population may not have been accurately estimated. Another hypothesis for the similarity between GBBP and GB0 could be because the base population that make up the composite breeds is very wide from a range of breeds and therefore, the allele frequencies from the base population estimated here may not reflect well the true allele frequency of the base population. Despite these assumptions, a previous study by Forni et al. [36] also suggested that similar results could be obtained using the allele frequencies from the current population. Based on that, we conclude that the observed allele frequencies (as in GB0) can be used for genomic predictions in this population.

The other scenario investigated was fitting **A** and **G** matrices in the mBVs estimation models (GB10 and GB20). The reason for that was to capture polygenic effects that were not captured by the markers. In beef cattle, also genotyped with HD SNP chip, Neves et al. [28] observed greater accuracies for some traits when fitting 20% of **A** (i.e. **GB20**). For the gestation length the authors observed an increase of 12% in accuracy. This trend was not observed in our study. The small differences seen between GB0, GB10 and GB20 are probably due to the density of the current SNP chip, which seems to be adequate in capturing most of the additive genetic variance for the traits in this population. Another reason for the small differences in our study could be due to pedigree incompleteness (dams were not recorded in two of the progeny test flocks). Similar to our results, Daetwyler et al. [5] and Aguilar et al. [37] have reported small increases in mBV accuracies when adding a polygenic effect into the model. Therefore, we do not recommend fitting **A** matrix as an option to increase accuracies under similar circumstances to our study.

#### Adjusting for population structure

The next strategy evaluated was to account for population structure by fitting PCs of **G** matrix as co-variables. The reason for the reduced accuracies when also fitting PCs could be because the population under study is composed mostly of crossbred animals or animals from composite breeds that share haplotypes among themselves and correcting for population structure may remove genetic effects that are important for the accuracy of genomic predictions. As discussed in Brito [11], several breeds were used in the development of these composites and some of them overlapped, which could explain in part their genetic connectedness.

The practice of adjusting for principal components to account for population structure has been reported in other sheep genomics studies [9, 18]. Similar results to those presented here, were reported by Daetwyler et al. [38] whom evaluated the effects of fitting a range of PC covariates (from one to 200) for greasy fleece weight and eye muscle depth measured in Australian sheep. The authors reported that the accuracy of genomic predictions clearly declined as an increased number of PCs were fitted.

Dodds et al. [18] investigated the effects of fitting PCs in genomic predictions of a New Zealand dual-purpose sheep population. The authors reported that the accuracies dropped by 0.02 between GB0 and GB6PC, which is much smaller than the reduction observed in our study. Therefore, the authors recommended to fit six PCs to take account of any spurious associations. Dodds et al. [18] also evaluated the changes in accuracies when adding the effects of PCs back into the estimates of mBVs. They observed that adding back PC effects does not have any advantage over fitting zero or a few PCs. The same trend was observed in this study (data not shown). The lowest correlations between GB0 and GB2PC, GB4PC and GB6PC observed for traits related to carcass fatness such as CGRM is probably due to more expressive differences among some of the composites (i.e. Primera composite presents larger range of carcass fatness compared to other breeds). As fitting PCs reduced considerably the accuracies of genomic predictions for the majority of the traits, we do not recommend fitting PCs when performing genomic predictions in a composite population, where the training and selection populations have a similar genetic structure or share ancestral breeds.

#### Cross-validation scenarios

Cross-validation can be useful in the case where the genetic composition of the animals in each year may vary. For example, if a producer of breed A decided not to genotype their animals in a specific year, it could influence the accuracy of genomic predictions for the other breed groups. It can also be useful when the selection candidates were born in a range of birth years and there are not many young animals (selection candidates) genotyped. When the subset of animals for cross-validation were randomly defined, the accuracies were higher than all other scenarios. It is due to a higher relationship among training and validation populations. Similar results were reported in the literature. For instance, Daetwyler et al. [5] when investigating genomic predictions for carcass and meat quality traits in a multi-breed population.

The next cross-validation approach (GBKCV) was defined based on k-means clustering. The objective of GBKCV validation design was to evaluate the prediction accuracies of genomic breeding values using a training population more distant to the selection candidates as pointed out by Saatchi et al. [32]. In practice it could happen if some producers from specific breeds decide not to genotype animals in some years, it could change the genetic structure of the training population and consequently decrease the accuracies of genomic predictions. Another possibility could be if there is a producer who started to genotype a breed (or different population), which has not been genotyped before and is less genetically related to the composite population under investigation. Our findings showed that in this case the accuracies (GBKCV) would be lower than those for the other scenarios, but it would still be possible to perform genomic selection with a reasonable level of accuracy for most traits. The reason for the lower accuracies for GBKCV is because the animals belonging to each individual cluster were more closely related among themselves and more distantly related to the other clusters, which resulted in a lower relationship between training and validation populations, reflected in lower accuracies. Reductions in accuracy depended on the genetic composition of the animals from each cluster/validation group used as validation and those in the training, as also observed by Toosi et al. [39]. Saatchi et al. [32] working with data from American Angus beef cattle reported a similar trend where random clustering accuracies were markedly higher than those from k-means clustering, on average by 0.21. The higher values of accuracy obtained by random clustering and forward validation is due to the higher genetic relationship between the animals from training and validation populations.

#### Genomic predictions within k-means clusters (GBC) versus mixed training population (GB0)

To characterize a scenario where genomic predictions are performed within a genetically homogenous sub-group of all the animals as opposed to using a mixed training population, genomic predictions were firstly conducted within each k-means cluster (GBC). Instead of using k-means clustering, animals could alternatively be separated based on flocks or recorded breed composition. In this study, we decided to evaluate clustering based on genomic information as it would be a more accurate clustering approach due to the high admixture of breeds in this population. As presented in Fig. 1, the animals were not clustered in distinctly separated groups, indicating that the majority of the animals are genetically related to some extent, hence the GB0 (mixed training population) resulted in higher accuracies of genomic predictions compared to GBC. As the animals are related, doing predictions within cluster is only reducing the size of training population. As reported in the literature, the calculation of mBVs depends, among other factors, on the size of the training population and the extent of the LD between SNP and QTL [25, 40–44]. As shown in Brito [11], this population presented a high enough level of LD to successfully perform genomic selection. However, the relatively small training population for some groups (genomic clusters) and the low heritability of some traits (Fig. 3) may be the reasons for the reduced accuracies of mBVs under GBC method. Therefore, a mixed training population is more beneficial. In a practical situation where the breeders had only one (or few) of the groups (clusters) to perform genomic selection, they would need to genotype more animals to increase the accuracies of genomic predictions of mBVs. Both the size of the training population and the number of animals in the validation are limiting factors for achieving reasonable high accuracies. In this study, validation groups with few animals (<150) were excluded from the mBV accuracy estimation.

Benefits of multi-breed genomic predictions have also been reported in other studies [42, 45–47]. Hozé et al. [48] working with three dairy cattle breeds and HD SNP chip (777 K) also observed that multi-breed GS can contribute to increased genomic evaluation accuracy in small breeds (or populations). Pryce et al. [49] in a study with three cattle breeds (Fleckvieh, Holstein, and Jersey) observed minimal advantage of multi-breed genomic evaluations over single-breed evaluations. However, when the goal was to predict genomic breeding values for a breed with no individuals in the training population, using two other breeds in the training was generally better than only one breed. It suggests that for small breeds or populations, mixed training populations can be very advantageous.

#### Genomic clustering based on G and EDM matrices (K5EDM, K5G, K10G and K10EDM) versus mixed training population (GB0)

Adding information from unrelated breeds to the training population could have no impact on the resulting mBV accuracies. However, the effect could also be negative, as marker effects may be averaged across breeds and marker allele frequencies may differ between breeds [10]. In beef cattle, Ventura et al. [41] reported increased accuracy when the training population was defined based on genomic clustering methodologies and no animals from different clusters were included. In this study we also investigated the same approach. However, no gains in accuracy were observed. One of the reasons is because the majority of the animals were clustered together and the exclusion of a few less related animals was not enough to impact the accuracies of genomic predictions. This confirms that within this dataset, genomic predictions are best derived using a mixed training population and excluding some less related animals did not result in improvements in mBV accuracies.

Moghaddar et al. [10] compared the accuracies of genomic predictions in purebred and crossbred Australian sheep using a 50 K SNP chip. The authors concluded that using data from distant breeds in the training population caused zero to small negative effects on genomic prediction accuracies, suggesting that when using the 50 K SNP chip a breed-specific training population is preferred. However, in the present study we used a HD SNP chip, which seemed to be more appropriate to conduct genomic predictions in a Terminal Sire composite population with high levels of genetic diversity [11], genetic connectedness (Fig. 1) and similar gametic phase of LD between SNP and causal mutations or QTLs [11].

#### Genomic predictions using crossbred data

In our study, animals from Terminal Sire composite breeds or Texel were selected based on crossbred (crossed with maternal/dual-purpose breed dams) progeny data. There was no available information on purebred (Terminal x Terminal) animals for comparisons. However, there are other studies in the literature in this regard. Moghaddar et al. [10] have reported that information from crossbreds of the target breed can be used in genomic prediction of purebred animals. Grevenhof and van der Werf [50] using a simulated pig dataset evaluated the benefits of including various proportions of crossbred animals in a training population for genomic selection of purebred animals in a crossbreeding program. The authors concluded that using crossbred rather than purebred data in a training population for genomic selection can also provide substantial advantages. In a simulated study, Esfandyari et al. [51] observed that training on crossbred animals yielded a larger response to selection in crossbred offspring compared to training on both pure lines separately or on both pure lines combined into a single training population. They also concluded that response to selection in crossbreds was greater when both phenotypes and genotypes were collected on crossbreds, compared to having only phenotypes on the crossbreds and genotypes on their parents.

### Spread of molecular breeding values

Most studies of genomic predictions in dairy cattle report the slope of EBV (based on extensive progeny testing) regressed on the mBV as a measure of genomic inflation. In sheep populations accuracies are generally not as high as those observed in dairy cattle. Therefore, *K* values are estimated as a measure of genomic inflation [18]. The expected value was 1, which would indicate that genomic predictions are on a similar scale as the phenotypes, i.e. not inflated or deflated. Values smaller than 1 indicate that the mBVs are more spread than expected and values greater than 1 are less spread than expected. Dodds et al. [18] proposed multiplying the raw mBVs by these *K* values to get them back to the expected spread before reporting them to producers to be used for selection.

The variation in scale observed in this study may be due to differences inherited to the data analyzed (e.g. the extent to which training animals were pre-selected) as pointed out by Neves et al. [28]. However, the *K* values observed in this study are similar to what we expected when using adjusted phenotypes and are in agreement with results reported in the literature. Dodds et al. [18] reported *K* values ranging from 0.16 to 0.90. Slopes well different from 1 have been reported in other studies [28, 45, 52, 53].

Even though the inclusion of polygenic effect did not increase the accuracy of mBVs, a slight improvement in the spread of mBVs was observed. A similar trend was also reported by Hozé et al. [48]. We believe that reporting *K* values are important for the scaling of mBVs before reporting it to breeders.

### Commercial implications

In this study we report results from a comprehensive analysis of genomic selection across several economic traits for Terminal Sire composites and using a HD SNP chip. The prediction equations developed will allow genomic selection to be applied in New Zealand Terminal Sire composites and crossbreds for various growth, carcass and meat quality traits. This will make it possible to select rams and ewes at an earlier age for breeding, thus reducing both generation interval and the cost of keeping lambs until their progeny are evaluated. It also allows for a higher selection intensity at birth and allows differentiation between full sibs, as multiple bearing ewes are frequent in sheep. Although the generation interval in sheep is not as long as in cattle it can still play a role for carcass and meat quality traits that are measured post-mortem. The statistics ENP (Tables 2, 3 and 4) indicates the number of progeny with phenotypic information needed in order to achieve similar accuracy that would be achieved at an early age by using genomic information. It is also important to highlight for the industry, the need to maintain performance recording to continuously update the training population. As prediction ability is influenced by the number of training animals, prediction accuracy would also be expected to increase over time.

## Conclusions

The accuracies reported in this study support the feasibility of genomic selection for growth, carcass and meat quality traits in New Zealand Terminal Sire breeds using the HD SNP chip. Our findings indicate that relatively accurate mBVs can be estimated for various traits at an earlier age of the lamb’s life and be used for selection, saving costs with progeny testing and reducing generation interval. It will be more beneficial for traits such as carcass and meat quality traits that are difficult and expensive to measure and in general can only be performed post-mortem.

There was a clear advantage to using a mixed training population instead of performing analyzes per genomic clusters. In order to perform genomic predictions per group, genotyping more animals is recommended in order to increase the size of the training population. Other alternative to increase the size of the training population is to share genotypes and phenotypes (EBVs) with other institutions/countries which may have data for genetically similar breeds. The different scenarios evaluated in this study will help geneticists and breeders to make wiser decisions in their breeding programs.

## Additional files

Additional file 1: Fixed effects included in the traits adjustment. (DOCX 13 kb)
Additional file 2: Number of individuals in the training and validation sets per trait for each genomic prediction scenario. (XLSX 22 kb)
Additional file 3: Pearson correlations between mBVs estimated using adjusted phenotypes (not including PCs, GB0) and phenotypes also adjusted for 2, 4 or 6 PCs (GB2PC, GB2PC, GB2PC, respectively). (DOCX 14 kb)
Additional file 4: Accuracies of GBLUP predictions for animals clustered based on G or EDM matrices. (XLSX 20 kb)

## Abbreviations

BLUP: Best Linear Unbiased Predictions
CG: Contemporary group
DNA: Deoxyribonucleic acid
EBV: Estimated breeding value using phenotypic information only (no marker information)
EDM: Euclidean Distance Matrix
ENP: Effective number of progeny
G: Genomic relationship matrix
GBLUP: Genomic Best Linear Unbiased Predictions
GS: Genomic selection
HD: High density
ISGC: International Sheep Genomics Consortium
L: Genome length
LD: Linkage disequilibrium
MAF: Minor allele frequency
mBV: Molecular breeding value (i.e. breeding value predicted from marker information)
N_e_: Effective population size
PC: Principal components
QTL: Quantitative trait loci
REML: Restricted maximum likelihood
SD: Standard deviation
SIL: Sheep improvement limited
SNP: Single nucleotide polymorphism
TBV: True breeding value
