# Supplementary material for: Prediction of genomic breeding values for growth, carcass and meat quality traits in a multi-breed sheep population using a HD SNP chip
Source: BMC Genet. 2017 Jan 26;18:7. doi: 10.1186/s12863-017-0476-8 (PMC5267438; doi:10.1186/s12863-017-0476-8)
Supplement: Additional file 1: — Fixed effects included in the traits adjustment. (DOCX 13 kb) [file 12863_2017_476_MOESM1_ESM.docx]

| **Table S1.** Fixed effects included in the traits adjustment. | | |
| --- | --- | --- |
| **Trait^1^** | **Fixed effects** | **Co-variables** |
| BWT | Sex, CG |  |
| WWT | Sex, CG | bdev |
| LW6 | Sex, CG | bdev |
| PRESLT, CWT, CWTC, DO% | Sex, CG | bdev |
| EMD, EMW, FDM | Sex, CG | bdev |
| EMDad, EMWad, FDMad | Sex, CG | bdev, LW6 |
| CBUTT, LEGWT, LEGLGTH, LNBNWT, CGRM, TENLWT, TENLL | Sex, CG | bdev |
| CBUTTad, CGRMad | Sex, CG | bdev, CWT |
| SFXWT, SFLEG, SFMID, SFFORE | Sex, CG | bdev |
| LPH | Sex, CG |  |
| LPHad | Sex, CG | CWT |
| MARB | Sex, CG | bdev |
| MARBad | Sex, CG | CWT, bdev |
| SHF | Sex, CG | bdev |
| SHFad | Sex, CG | CWT, pH, pH^2^ |
| A24, A48, A96, A168, B24, B48, B96, B168, L24, L48, L96, L168 | Sex, CG | bdev |
| A24ad, A48ad, A96ad, A168ad, B24ad, B48ad, B96ad, B168ad, L24ad, L48ad, L96ad, L168ad | Sex, CG | CWT, bdev, pH |
| ^1^: See abbreviations on Table 1; CG: contemporary group for each trait was defined by flock, birth year, sex, weaning mob (except for birth weight) and trait measurement/slaughter mob; bdev: birthday deviation; | | |
