# Supplementary material for: Prediction of genomic breeding values for growth, carcass and meat quality traits in a multi-breed sheep population using a HD SNP chip
Source: BMC Genet. 2017 Jan 26;18:7. doi: 10.1186/s12863-017-0476-8 (PMC5267438; doi:10.1186/s12863-017-0476-8)
Supplement: Additional file 3: — Pearson correlations between mBVs estimated using adjusted phenotypes (not including PCs, GB0) and phenotypes also adjusted for 2, 4 or 6 PCs (GB2PC, GB2PC, GB2PC, respectively). (DOCX 14 kb) [file 12863_2017_476_MOESM3_ESM.docx]

| **Table S3.** Pearson correlations between mBVs estimated using adjusted phenotypes (not including PCs, GB0) and phenotypes also adjusted for 2, 4 or 6 PCs (GB2PC, GB4PC, GB6PC, respectively). | | | |
| --- | --- | --- | --- |
| **Trait^1^** | **GB0 x GB2PC** | **GB0 x GB4PC** | **GB0 x GB6PC** |
| **A24** | 0.99 | 0.98 | 0.98 |
| **A48** | 0.99 | 0.96 | 0.96 |
| **A96ad** | 0.99 | 0.98 | 0.97 |
| **B96** | 0.99 | 0.98 | 0.98 |
| **B96ad** | 0.99 | 0.98 | 0.98 |
| **B168ad** | 0.99 | 0.92 | 0.91 |
| **DRESS** | 0.99 | 0.93 | 0.92 |
| **EMW** | 0.99 | 0.97 | 0.97 |
| **EMWad** | 0.99 | 0.98 | 0.98 |
| **L96ad** | 0.99 | 0.98 | 0.97 |
| **SFRIB** | 0.99 | 0.99 | 0.99 |
| **WWT** | 0.99 | 0.95 | 0.95 |
| **A24ad** | 0.98 | 0.97 | 0.97 |
| **A48ad** | 0.98 | 0.96 | 0.96 |
| **A96** | 0.98 | 0.97 | 0.95 |
| **B24** | 0.98 | 0.96 | 0.96 |
| **B48** | 0.98 | 0.96 | 0.96 |
| **B48ad** | 0.98 | 0.96 | 0.95 |
| **B168** | 0.98 | 0.95 | 0.94 |
| **BWT** | 0.98 | 0.97 | 0.97 |
| **L24ad** | 0.98 | 0.98 | 0.97 |
| **L48ad** | 0.98 | 0.97 | 0.97 |
| **SHF** | 0.98 | 0.95 | 0.94 |
| **SFFORE** | 0.98 | 0.92 | 0.90 |
| **B24ad** | 0.97 | 0.96 | 0.95 |
| **CBUTT** | 0.97 | 0.91 | 0.90 |
| **EMD** | 0.97 | 0.93 | 0.94 |
| **EMDad** | 0.97 | 0.94 | 0.94 |
| **L24** | 0.97 | 0.96 | 0.97 |
| **L168ad** | 0.97 | 0.96 | 0.95 |
| **LW6** | 0.97 | 0.95 | 0.94 |
| **CBUTTad** | 0.96 | 0.90 | 0.87 |
| **CCWT** | 0.96 | 0.91 | 0.89 |
| **L48** | 0.96 | 0.96 | 0.95 |
| **L96** | 0.96 | 0.96 | 0.96 |
| **L168** | 0.96 | 0.98 | 0.94 |
| **SHFad** | 0.96 | 0.95 | 0.93 |
| **LPH** | 0.96 | 0.94 | 0.94 |
| **LPHad** | 0.96 | 0.95 | 0.94 |
| **SFXWT** | 0.96 | 0.91 | 0.90 |
| **PRESLT** | 0.95 | 0.93 | 0.92 |
| **SFLEG** | 0.95 | 0.90 | 0.90 |
| **A168** | 0.94 | 0.94 | 0.93 |
| **A168ad** | 0.94 | 0.94 | 0.93 |
| **HCWT** | 0.94 | 0.93 | 0.91 |
| **FDM** | 0.93 | 0.94 | 0.93 |
| **LMARB** | 0.93 | 0.93 | 0.92 |
| **LMARBad** | 0.93 | 0.93 | 0.92 |
| **FDMad** | 0.92 | 0.93 | 0.93 |
| **SFMID** | 0.90 | 0.90 | 0.90 |
| **CGRM** | 0.80 | 0.80 | 0.80 |
| **CGRMad** | 0.76 | 0.75 | 0.75 |
| ^1^Abbreviations are presented on Table 1. | | | |
